# Current look at the most promising proteomic and glycomic biomarkers of bladder cancer

**DOI:** 10.1007/s00432-024-05623-7

**Published:** 2024-02-19

**Authors:** Stefan Harsanyi, Kristina Kianickova, Jaroslav Katrlik, Lubos Danisovic, Stanislav Ziaran

**Affiliations:** 1 Institute of Medical Biology, Genetics and Clinical Genetics, Faculty of Medicine, Comenius University, Bratislava, Slovakia; 2 Institute of Chemistry, Slovak Academy of Sciences, Bratislava, Slovakia; 3 Department of Urology, Faculty of Medicine, Comenius University, Bratislava, Slovakia

**Keywords:** Bladder cancer, Diagnostics, Biomarkers, Protein, Glycoprotein

## Abstract

**Background:**

Bladder cancer (BC) belongs to the most frequent cancer types. The diagnostic process is still long and costly, with a high percentage of false-positive or -negative results. Due to the cost and lack of effectiveness, older methods need to be supplemented or replaced by a newer more reliable method. In this regard, proteins and glycoproteins pose high potential.

**Methods:**

We performed an online search in PubMed/Medline, Scopus, and Web of Science databases to find relevant studies published in English up until May 2023. If applicable, we set the AUC threshold to 0.90 and sensitivity/specificity (SN/SP) to 90%.

**Findings:**

Protein and glycoprotein biomarkers are a demonstrably viable option in BC diagnostics. Cholinesterase shows promise in progression-free survival. BLCA-4, ORM-1 along with HTRA1 in the detection of BC. Matrix metallopeptidase 9 exhibits potential for stratification of muscle-invasive subtypes with high negative predictive value for aggressive phenotypes. Distinguishing non-muscle invasive subtypes benefits from Keratin 17. Neu5Gc-modified UMOD glycoproteins pose potential in BC diagnosis, while fibronectin, laminin-5, collagen type IV, and lamprey immunity protein in early detection of BC.

## Introduction

Bladder cancer (BC) belongs to the top ten most common cancers worldwide, with over 200,000 yearly new cases in the European Union (EU) (EAU Guidelines 2023; Sung et al. 2021). Globally, BC is more frequent in males and also depends on the population and region. The American Cancer Society (ACS) reported that 90% of BC cases are in patients over 55 years of age, with an average age of 73.

At the time of diagnosis, the tumor can be characterized as non-muscle invasive bladder cancer (NMIBC) or muscle-invasive bladder cancer (MIBC), while about 90% of these tumors are of urothelial origin (Kaseb and Aeddula 2023). Diagnosis, therapy, and prognosis of NMIBC suffer from an unpredictable recurrence rate and a chance of progression, while MIBC often represents aggressive neoplasms with peripheral invasion, metastases, and poor response to treatment (Cho et al. 2009; Fukumoto et al. 2016). Classic immunohistochemical analysis of cancer tissue is widely utilized, but studies show that this method is unsuitable for the use of prediction of recurrence and progression (Bevizova et al. 2013; Repiska et al. 2010). Over the last few years, the European Association of Urology (EAU) has slowly adopted and implemented molecular markers into BC diagnosis, but they are not yet suitable for routine application, and cystoscopy in combination with cytology is still the gold standard (EAU Guidelines 2020, 2021, 2022).

This year, a new test for the prediction of future BC has been presented. The UroAmp test was effective in predicting 66% of future BCs in urine samples collected 12 years ago, at least 6 years before clinical diagnosis. The test was truly negative in 94 of the 98 participants (96%) who would not develop BC in the future. Mutations were found in 71% of urine samples in patients with already visible tumors. Mutations were not found in 94% of patients with a negative cystoscopy (EAU Guidelines 2023; Medscape 2023). Although not yet officially approved by the EAU or the American Urological Association (AUA), the specificity (SP) of 94% is a significant boost over the lower SN of already approved tests (e.g., BTA stat/TRAK, NMP22, Cell Search, UroVysion, uCyt +) at least except for the recently approved Uromonitor with specificity/sensitivity (SP/SN) of 73.5/93.2% (Lee et al. 2023).

In this review, we focused on published proteomic, and glycomic molecular markers associated with BC. The selection of proteomic biomarkers was based on the reported high sensitivity and specificity threshold. Finding new viable and more accurate biomarkers that could be used in screening will significantly enhance the diagnostic process. It will also supplement conventional diagnostic methods such as urine cytology and cystoscopy, with a future possibility to even replace them.

## Materials and methods

We performed an online search in PubMed/Medline, Scopus, and Web of Science using the terms “bladder cancer”, in combination with “glycomic”, “glycoprotein”, “proteomic”, “protein” and “markers” or “biomarkers” published up until May 2023. If applicable, we set the threshold of sensitivity and specificity (SN/SP) to be at least 90% for primary markers; while for panel markers, at least one variable needed to be over 90%. Panel markers are specified as possible synergic diagnostic biomarkers, that could enhance each other’s potential in a diagnostic panel.

Taking ground in the recent knowledge, we do not prefer the notion of an independent prognostic marker, since every marker and test has shown flawed results depending on sample preparation, transportation, or examination. Test results are also affected by inter- and intra-individual variabilities, such as the presence of a urologic condition or genetic variation.

The threshold of SN/SP is mainly used in proteomic markers with the addition of the area under the curve (AUC) score, positive predictive value (PPV), and negative predictive value (NPV), which were equally set to 0.90 and more, to ensure high diagnostic potential. As genes and various RNA molecules are usually not evaluated by SN/SP or AUC, the threshold of inclusion has been set on a repeated statistical significance reported by researchers.

## Proteomic biomarkers

As conventional methods face limitations, the comprehensive analysis of proteomics emerges as a beacon of hope for overcoming diagnostic challenges. Discussed proteomic biomarkers are presented in Table 1.

**Table 1:** Discussed proteomic and glycomic markers

Biomarker	Sample and collection	Encoding gene and cytogenetic location	Deviation from normal levels	References
Cholinesterase	Serum, pre-surgery	ACETYLCHOLINESTERASE; ACHE-7q22.1	↓	Kimura et al. (2018)
FGF21 and FGF23	Serum, pre-surgery	FIBROBLAST GROWTH FACTOR 21; FGF21-19q13.33 FIBROBLAST GROWTH FACTOR 23; FGF23-12p13.32	↑	Li et al. (2019)
Cytokeratin 19 fragment	Serum, urine	KERATIN 19; KRT19-17q21.2	↑	Huang et al. (2015)
Annexin V and S100A9	Serum, pre-/post-surgery	ANNEXIN A5; ANXA5-4q27 S100 CALCIUM-BINDING PROTEIN A9; S100A9-1q21.3	↑	Bansal et al. (2016)
S100A8 and S100A9	Serum, pre-/post-surgery	S100 CALCIUM-BINDING PROTEIN A8; S100A8-1q21.3	↑	Yasar et al. (2017)
HNRNPA3	Cancer tissue, post-surgery	HETEROGENEOUS NUCLEAR RIBONUCLEOPROTEIN A3; HNRNPA3-2q31.2	↑	Amano et al. (2021)
Dimethyl amine, glutamine, lactate, histidine, and valine	Serum, pre-/post-surgery	–	↑	Gupta et al. (2020)
MMP-9	Urine and blood	MATRIX METALLOPROTEINASE 9; MMP9-20q13.12	↑	Candido et al. (2016), Chang et al. (2020), Liu et al. (2020)
Alcohol and aldehyde dehydrogenase	Serum	ADH Class I-ADH1A, ADH1B, ADH1C at 4q23 ALDH—12 genes, multiple locations	↑	Orywal et al. (2017, 2018)
Survivin	Serum and urine	BACULOVIRAL IAP REPEAT-CONTAINING PROTEIN 5; BIRC5-17q25.3	↑	Jazayeri et al. (2020)
HAS (HAS1, HAS2, HAS3)	Cancer tissue, post-surgery	HYALURONAN SYNTHASE 1; HAS1-19q13.41 HYALURONAN SYNTHASE 2; HAS2-8q24.13 HYALURONAN SYNTHASE 3; HAS3-16q22.1	↑	Adamia et al. (2005), Itano et al. (2004), Golshani et al. (2007), Golshani et al. (2008), Kramer et al. (2011)
HYAL-1	Cancer tissue, urine	HYALURONOGLUCOSAMINIDASE 1; HYAL1-3p21.31	↑	Eissa et al. (2005), Kramer et al. (2010), Mammadov et al. (2014), Dong et al. (2021)
BLCA-4	Cancer tissue, urine	BLCA-4	↑	Lotan and Roehrborn (2003), Shirodkar and Lokeshwar (2009), Konety et al. (2000) Feng et al. (2011), Cai et al. (2015), Alavi et al. (2019)
ORM1	Urine	OROSOMUCOID 1; ORM1-9q32	↑	Hou et al. (2014), Li et al. (2016)
HTRA1	Cancer tissue	HTRA SERINE PEPTIDASE 1; HTRA1-10q26.13	↓	Clausen et al. (2002), Shridhar et al. (2002), Baldi et al. (2002), Chien et al. (2004), Esposito et al. (2006), Lorenzi et al. (2013), Altobelli et al. (2018)
Keratin 17	Urine	KERATIN 17, TYPE I; KRT17-17q21.2	↑	Vasdev et al. (2021), Babu et al. (2019, 2021), Li et al. (2021)

Cholinesterase (ChE) is a predictor of progression-free survival (PFS). ChE is a soluble enzyme in human plasma and serum. Human blood contains ten times more butyrylcholinesterase (BuChE) than acetylcholinesterase (AChE). The average human serum contains 4–5 mg BuChE per liter, but only 0.5 mg AChE per liter of blood. A retrospective study on 1117 NMIBC patients by Kimura et al., reported a 5-year PFS in patients with low and normal ChE levels were 93.2% and 91.4%, respectively (*p* = 0.053). ChE was significantly associated with shorter recurrence-free survival (RFS). ChE was also a strong predictor of disease recurrence (Kimura et al. 2018).

Li et al. analyzed the circulating FGF19, 21, and 23 levels in 67 patients. All median serum levels of BC patients deviated from healthy controls. All were significant predictors of BC, and ROC (receiver operating characteristic) curves with AUC—0.674 (*p* = 0.015); AUC—0.918 (*p* < 0.001); AUC—0.897 (*p* < 0.001), respectively. FGF21 and FGF23 show promise, although mainly missing the SN/SP threshold, save for the specificity of 96.43% for FGF23. FGF21 (*p* = 0.025) was increased in patients with a history of recurrence (Li et al. 2019).

In a meta-analysis by Huang et al., cytokeratin 19 fragment (CYFRA 21-1) pooled SN from urine and serum were 82% and 42%, respectively. The pooled SP was 80% and 94%, respectively (Huang et al. 2015). A recent meta-analysis by Zhu et al. did not differentiate the source of the marker between serum and urine. The reported pooled SN/SP of 69/81% with AUC—0.89 is a confirmation of the previous meta-analysis. Based on current knowledge we can conclude that CYFRA 21-1 is not suitable as an independent prognostic or diagnostic marker, although the SN/SP in urine could contribute to a combined urine protein panel.

Calcium ion-binding proteins S100A8 (MRP8) and S100A9 (MRP14), along with S100A4, CA1 (carbonic anhydrase 1), and Annexin V (annexin 5) proteins were studied by Bansal et al., with reported significant differences in pre-and post-operative levels in BC patients (Bansal et al. 2016). Namely, S100A9 (AUC—0.957; SN 92.3%; SP 83.6%) and Annexin V (AUC—0.921; SN 90.4%; SP 80.0%) showed the greatest promise, however, Yasar et al. reported no difference between serum levels of S100A8 and S100A9 in the study or control group (Yasar et al. 2017). These markers show promise in research of prostate cancer (Novakova et al. 2014; Grebhardt et al. 2014). In the later study, Calprotectin serum levels were significantly elevated in the whole study group if compared to controls, although without the possibility of stratifying patients based on stage or grade. Urine calprotectin levels were significantly higher in the study group, with an additional two- to threefold elevation in MIBC (T2–4) compared to NMIBC (Ta and T1).

Urine BTA (bladder tumor antigen) was higher in the study group, but due to the long intervals of chosen stage/grade groups unviable for patient stratification (Yasar et al. 2017).

A recent study by Amano et al., on the heterogeneous nuclear ribonucleoprotein A3 (HNRNPA3) expression, was shown to be significantly associated with lymph node metastasis and S100A8, S100A9, and uroplakin III expressions (Amano et al. 2021).

A pre- and post-operative evaluation of targeted serum metabolic biomarkers by Gupta et al., reported AUC of 0.868–0.934, with SN/SP of around 80% for dimethyl amine (DMA), glutamine, lactate, histidine, and valine. While all missed the 90% threshold, these serum biomarkers could be used in a combined serum pallet (Gupta et al. 2020).

The matrix metallopeptidase 9 (MMP-9) was studied by Candido et al., where the higher urinary levels of NGAL, MMP-9, and NGAL/MMP-9 complex were recorded in the group of current smokers, but no dietary impact was observed. Significant elevations occurred in MIBC (T2–T4) and non-papillary BCs. ROC analysis proposed MMP-9 as a viable marker (AUC—0.68), but for MIBC, the performance was significantly better (AUC—0.90) with NPV of 97% for aggressive phenotypes of BC (Candido et al. 2016). The participation of the MMP9 axis was lately studied in association with miRNA and circRNA, where inhibition of MMP9 protein degradation by miRNA-516a promoted cancer metastasis, and MMP9 upregulation in circ0001361-overexpressed BC cells promoted cell migration and invasion (Chang et al. 2020; Liu et al. 2020).

The study of Orywall et al. on serum ADH (alcohol dehydrogenase) isoenzymes and ALDH (aldehyde dehydrogenase) activity found a significantly higher total activity of ADH in sera of both, low-grade and high-grade BC patients, with ADH activity (AUC—0.848; SN 81.5%; SP 98.1%; PPV 97.4%; NPV 92.3%) (Orywal et al. 2017, 2018).

Survivin SN/SP performance in research  is reported in intervals of 60–80%, however novel approach by Jazayeri et al., using the survivin antibody-conjugated gold nanoparticles (GNPs) dependent on visible color changes and colorimetric detection (reader devices not needed), allowing for the protein to be detected in the urine of patients with both LG and HG tumors. By this method, LG tumors could be diagnosed by visible color changes and colorimetric detection (Jazayeri et al. 2020).

Hyaluronic acid (HA) is synthesized at the plasma membrane by any one of the three hyaluronan synthases (HAS). HAS1, HAS2, and HAS3 are transmembrane proteins, which synthesize different sizes of HA at different kinetic rates (Adamia et al. 2005). HAS1 and HAS2 expression increases following malignant transformation and promotes tumor growth (Itano et al. 2004). The expression of HAS1 was studied in BC in relation to the urinary HA test (Golshani et al. 2007). The study demonstrated that HAS1 type HA-synthase is expressed in BC cells and the expression is upregulated, both at the transcriptional and translational levels, suggesting that HAS1 expression may have prognostic potential related to tumor recurrence and progression. Similar works showed that HAS1 modulates HA and CD44 levels, affecting tumor growth and progression in BC, and HAS1 mRNA expression was associated with BC metastasis (Golshani et al. 2008; Kramer et al. 2011).

Hyaluronidase-1 (HYAL-1) is the major hyaluronidase expressed in BCa cells and is an accurate marker for high-grade BC (Eissa et al. 2005). High expression of Hyal1 has been suggested to predict MIBC and recurrent BC and is associated with metastasis and decreased disease-specific survival (Kramer et al. 2010). However, in pT1 BC, the expression of HYAL-1 did not have any prognostic importance (Mammadov et al. 2014). In the recent meta-analysis, HYAL-1 demonstrated the highest PPV. In terms of accuracy indicators, HYAL-1, UCA1, and survivin had the highest accuracy concluding that HYAL-1 and survivin are suitable urine biomarkers for BC diagnosis. This ranks HYAL-1 as the most promising urine biomarker (Dong et al. 2021).

BLCA-4 is a member of the nuclear matrix protein family and is involved in tumor cell proliferation, survival, and angiogenesis (Lotan and Roehrborn 2003). BLCA-4 was reported to be the most sensitive and specific urinary marker with an SN range from 89% to 96.4% and an SP range from 95% to 100% (Shirodkar and Lokeshwar 2009; Konety et al. 2000; Feng et al. 2011). A meta-analysis summarized the pooled SN of 0.93 (95% CI, 0.90–0.95) and the pooled SP of 0.97 (95% CI, 0.95–0.98), which represent a promising diagnostic marker in BC (Cai et al. 2015). Alavi et al. reported significantly higher BLCA-4 urine levels in patients with BC (*p* < 0.001), while tumor stage and size showed no association (Alavi et al. 2019).

ORM1 (Orosomucoid-1) plays an important role in modulating the activity of the immune system during acute-phase reactions and other chronic diseases (Hou et al. 2014). Urinary ORM1 levels were identified and significantly elevated in BC compared to healthy controls by proteomic methods, and an optimum cut-off value of 3912.97 ng/mg corresponding to 91.96% SN and 94.34% SP was identified. A cut-off value of 7351.28 ng/mg was utilized to distinguish infiltrating urothelial carcinoma from BC patients corresponding to 91.89% SN and 90.67% SP (Li et al. 2016).

HTRA1 (high-temperature requirement 1)—a member of the family of HtrA proteins, is a secreted multidomain protein with serine protease activity (Clausen et al. 2002). Changes in its expression associated with tumorigenesis have been reported, e.g., HtrA1 down-regulation has been described in human ovarian cancer (Shridhar et al. 2002). Also, its over-expression was detected to suppress the proliferation and migration of tumor cells in highly invasive melanoma (Baldi et al. 2002). In various solid tumors, such as ovarian and lung cancer the expression of HtrA1 is down-regulated (Chien et al. 2004; Esposito et al. 2006). In the study by Lorenzi et al. the SN/SP values calculated at a cut-off value of 1.48 (HtrA1), were 92.65% (C.I. 86.44–98.85%) and 95.59% (C.I. 90.7–99.7%), respectively. Highlighting a very good global performance of the diagnostic test (AUC is equal to 0.9839, C.I. 0.97–0.99) (PPV = 95.45% (C.I. 90.42–99.7%, NPV = 92.86%) (Lorenzi et al. 2013). Recently, a meta-analysis was conducted to assess the potential role of HtrA1 as a tumor marker and/or prognostic factor in several tumors (Altobelli et al. 2018). There was a statistically significant difference between the tumor samples and healthy controls, with a consistent finding of this work, that HtrA1 levels are higher in healthy controls or normal-looking tissue than in diseased tissue from patients with a variety of tumors. This implies that HtrA1 could be a promising biomarker in BC.

Keratin 17 (K17) is a type I cytokeratin found in various epidermal appendages such as hair follicles or sebaceous glands. The presence of K17 in the urine of patients with BC is the newest significant biomarker currently under study. The test kit is used under the name of Uro17™. Vasdev et al., reported SN/SP of 100%/92.6% for both NMIBC and MIBC detection (Vasdev et al. 2021). Babu et al. reported an SN of 89% with an SP of 88% for distinguishing between malignant and normal tissue in biopsies (Babu et al. 2019). Babu et al. also found that only five K17-positive cells were sufficient threshold for a positive test (AUC = 0.90), where SN ranged from 86% or 97%, the first for a biopsy-confirmed BC, the second in a discovery and validation cohort, while and SP of 100% for high-grade and 84% for biopsy-confirmed tumors (Babu et al. 2021). Elevated expression of K17 in BC cells on mRNA and protein levels was found in comparison with the normal human urothelial cells (Li et al. 2021).

## Glycomic biomarkers

Glycomic biomarkers comprise:

- Glycoproteins are presented in Table 2, of which only the first four are included for lack of information on the ones currently under investigation.
- Markers of glycosylation.

**Table 2:** Promising glycoproteins currently under investigation

Glycoprotein	Encoding gene	Exam. methods	Refs.
HOMER3	HOMER SCAFFOLD PROTEIN 3; HOMER3-19p13.11	MS	Peixoto et al. (2021)
Fibronectin, laminin-5, and type IV collagen	FIBRONECTIN 1; FN1-2q35 LAMININ, ALPHA-5; LAMA5-20q13.33 COL4A1-6; chromosomes 1, 2 and X	SPRi	Guszcz et al. (2023)
GANAB protein	GLUCOSIDASE, ALPHA, NEUTRAL AB; GANAB-11q12.3	IHC	Lin et al. (2022)
P4HB	PROCOLLAGEN-PROLINE, 2-OXOGLUTARATE-4-DIOXYGENASE, BETA SUBUNIT; P4HB-17q25.3	Gene expression	Wu et al. (2021)
Leukocyte differentiation antigen CD36	CD36 ANTIGEN; CD36-7q21.11	TMA, IHC	Jeong et al. (2021)
CD93 (C1QR1)	COMPLEMENT COMPONENT 1, q SUBCOMPONENT, RECEPTOR 1; C1QR1-20p11.21	Gene expression	Zheng et al. (2022)
Cartilage oligomeric matrix protein (COMP)	CARTILAGE OLIGOMERIC MATRIX PROTEIN; COMP-19p13.11	IHC, RT-qPCR	Kuo et al. (2022)
Neurexophilin 4	NEUREXOPHILIN 4; NXPH4-12q13.3	Gene expression, IHC	Sun et al. (2022)
Alpha-2-Heremans-Schmid Glycoprotein (AHSG)	ALPHA-2-HS-GLYCOPROTEIN; AHSG-3q27.3	Cell proliferation assay, WB, ELISA, IHC	Dong et al. (2022)
Fibulin-3	EGF-CONTAINING FIBULIN-LIKE EXTRACELLULAR MATRIX PROTEIN 1; EFEMP1-2p16.1	IHC	Al Khader et al. (2022)
MUC1	MUCIN 1, CELL SURFACE ASSOCIATED; MUC1-1q22	Gene expression	Qing et al. (2022)
Human leukocyte antigen, DR alpha chain (HLA-DRA)	MAJOR HISTOCOMPATIBILITY COMPLEX, CLASS II, DR ALPHA; HLA-DRA-6p21.32	Gene expression	Piao et al. (2021)
Tenascin-C	TENASCIN C; TNC-9q33.1	IHC, RT-qPCR, WB, …	Guan et al. (2022)
Vascular cell adhesion molecule-1 (VCAM-1)	VASCULAR CELL ADHESION MOLECULE 1; VCAM1-1p21.2	Quantitative immunoassay	Mori et al. (2022)
Thrombospondin-5 (TSP-5)	CARTILAGE OLIGOMERIC MATRIX PROTEIN; COMP-19p13.11	IHC	Harada et al. (2021)
Ribophorin II (RPN2)	RIBOPHORIN II; RPN2-20q11.23	Gene expression, WB, IHC	Han et al. (2021)

*MS* mass spectrometry; *SPRi* surface plasmon resonance imaging; *IHC* immunohistochemistry; *TMA* tissue microarray; *RT-qPCR* quantitative reverse transcription polymerase chain reaction; *WB* western blot; *ELISA* enzyme‐linked immunosorbent assay

### Glycoproteins

Glycoprotein HOMER3 can be found in BC at the cell surface covered with short-chain sialylated O-glycans and was identified as a potential predictor of the worst prognosis of BC, and its expression was hypothesized to be driven by hypoxia and glucose deprivation. Analyzed were BC cell lines of grade I–IV and 104 FFPE BC tissue samples. The result of the HOMER3 knockdown was a decrease in proliferation and on the opposite, after the HOMER3 knock-in there was an increase in its membrane expression. HOMER3 expression was not noticed in healthy urothelial tissue. Using mass spectrometry (MS) were also identified potentially important domains in HOMER3-glycosides of BC tissues. HOMER3-STn glycoforms look promising in the case of precise targeting for clinics (Peixoto et al. 2021).

Fibronectin, laminin-5, and type IV collagen are glycoproteins present in the urothelial basement membrane (UBM), and all bladder cancer staging over Ta (T1–T4) may demonstrate disturbances in the UBM structure, as well as alterations in the serum concentrations of UBM proteins. These proteins were analyzed using surface plasmon resonance imaging (SPRi) biosensors in 92 serum samples with different BC characteristics. Differentiation between controls and BC samples showed satisfactory results with AUC scores of 0.92–0.99. The sensitivity of the assay was 98–100% and the specificity was 62–92%, which can be a good predictor in the early detection of BC (Guszcz et al. 2023).

The poor prognosis of urothelial carcinoma relates to higher expression of Glucosidase II α-subunit (GANAB protein), which is a regulator of glycosylation that could be used as a prognostic biomarker for urothelial carcinoma. 107 FFPE samples and two cell lines were analyzed using immunohistochemistry (IHC) assay, and upregulated expression of GANAB was in correlation with high tumor grades, promoting proliferation, invasion, or migration of carcinoma cells. This protein can serve as a prognostic biomarker correlated with unfavorable outcomes of urothelial carcinoma (Lin et al. 2022).

Beta-subunit of prolyl 4-hydroxylase (P4HB) glycoprotein having also impact on N-glycan modification in endoplasmic reticulum was studied as a novel biomarker with diagnostic and prognostic value in diagnostic of BC. 69 pairs of BC and control tissues were analyzed, and the AUC value for expression of P4HB to discriminate between tumor and normal control tissues was up to 0.881 (95% CI: 0.825–0.937; *p* < 0.001) (Wu et al. 2021).

**Fig. 1:**
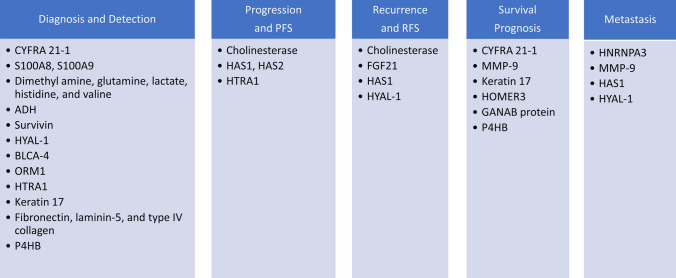
Association of proteomic and glycomic markers with BC diagnosis

In the study with 417 FFPE BC samples by IHC and bioinformatic tools, versican, a chondroitin sulfate proteoglycan, was identified as a prognostic marker upregulated in BC (Zhang et al. 2021). Other newly studied glycoproteins with a promising outlook for the characterization of grade, progression, or implementation of better-targeted therapy of BC are listed in Table 2, while their association with BC is presented in Fig. 1.

### Markers of glycosylation

The importance of glycomic biomarkers, biomarkers based on the detection of altered glycosylation, for early diagnosis, prognosis, and therapy of many diseases, including cancer, is growing rapidly, and this applies to BC as well. In recent years, there were several published reviews dedicated to the glycan and glycoprotein changes related to BC (Jian et al. 2020; Jin et al. 2021; López-Cortés et al. 2021; Wilson et al. 2021). In this section, we discuss the newest research on BC glycomic biomarkers that were reported during the last 2 years.

Glycosylation changes on glycoprotein CD44 were studied with 75 FFPE patients’ bladder tumor tissues (34 NMIBC, 41 MIBC), 11 control tissues, and 3 cell lines of BC by a combination of various approaches, glycomics including several types of immunoassays and double staining immunofluorescence, glycoproteomics and transcriptomic, for better and more complex characterization of samples. CD44-Tn/Sialyl-Tn (STn) glycoforms were identified as the important signatures of aggressiveness in the case of BC requiring targeted interventions (Gaiteiro et al. 2022).

Characterization and differences in glycosylation between low-grade BC, high-grade BC, and control epithelial cells were studied using various glycomics approaches. Cell lines containing cells with low-grade BC (RT4, 5637, SW780) showed higher expression of fucosylated Lewis X (LeX) antigen in comparison with control bladder cells of epithelium (A/T/N), while in the high-grade BC cell lines (J82COT, T24, TCCSUP), there was only a little or no expression of LeX reported. Some other differences in fucosylation and sialylation of N-glycans were identified between these three groups as well. Moreover, different expression of glycosyltransferases between low-grade and high-grade BC cell lines were related to different expression of O-glycans (Ezeabikwa et al. 2021). Determination of cells' surface truncated O-glycans in relation to more types of cancers including BC revealed that Tn and STn expression is decreasing with higher tumor grade (Rømer et al. 2021).

Analysis of BC urine biomarker, UMOD glycoprotein that is present in the urine of people with BC, and lamprey immunity protein (LIP), a lectin that specifically binds to multi-antennary sialylated N-glycolylneuraminic acid (Neu5Gc) structures on UMOD glycoproteins in the urine of BCa patients, was performed in ELISA format. The number of samples for this primary screening was over 3500 samples of different stages (BC, benign, controls). The AUC (95%) value was 0.9422 when the benign group was chosen as the control. The results indicate that Neu5Gc-modified UMOD glycoproteins present in urine and sialyltransferases may function as potential markers in clinical trials, and LIP may be a tool for early BCa identification, diagnosis, and monitoring (Teng et al. 2022).

The discrimination between urothelial carcinoma (UC), including BC and upper urinary tract UC (UTUC) was performed by studying N-glycans on immunoglobulin using gel electrophoresis (GE) on 104 BC, 68 UTUC, 62 controls, 10 urinary tract infections, and 5 cystitis serum samples. BC and UTUC scores for discriminating BC and UTUC were defined by AUC, 0.977 and 0.867 (Kodama et al. 2021). In another study, the diagnostic of 9 urological diseases, including BC, was performed by characterization of N-glycan signatures of immunoglobulin (Ig). 1213 serum samples, 176 of them from BC patients and 269 healthy volunteers were analyzed by CE and data were processed by machine learning to obtain characteristic Ig N-glycan signatures for these nine diseases. A combination of α-2,3 sialyl tetraantennary N-glycan and agalactosyl bisecting GlcNAc core fucosyl N-glycan was sufficient for specific detection of BC. The AUC and specificity at 90% sensitivity of the BC score versus seven diseases had a high value (0.99% and 98%, respectively), and urinary tract infection had a lower value (0.90% and 77.0%, respectively) (Iwamura et al. 2022).

Upregulation of glycosyltransferase C1GALT1 was found in serum and tissue samples from BC patients. Moreover, this transferase is responsible for the production of T antigen, which was also upregulated in high-grade BC (Tan et al. 2022).

A recent study refuted the previous claims about down-regulation in the expression of ST6GAL1 in the case of BC. The ST6GAL1 Golgi sialyltransferase is upregulated in many human malignancies, however, many commercial antibodies for ST6GAL1 do not, in fact, recognize ST6GAL1, what was the reason for this mistake? The samples were analyzed using tissue microarrays with IHC staining, and this result is in correlation with those obtained for other cancers (Haldar et al. 2022).

Disruption of N-glycan expression in pancreatic adenocarcinoma to reverse the resistance of tumor cells to be killed by chimeric antigen receptor (CAR) T cells was studied by two different approaches, ablation of MGAT5 (mannoside acetyl-glucosaminyltransferase 5) in tumor cells or treating of mice with 2-deoxy-d-glucose. The approach of treating with 2-deoxy-d-glucose with CAR T cells was also efficient for other types of cancer, including BC. It was found a negative correlation between the expression of N-glycans and the killing potency of CAR T cells can be considered a predictive biomarker in patients with ineffective CAR T cell therapy (Greco et al. 2022).

## Conclusion and future directions

There are a few new and notable markers, e.g., serum irisin, that in a new study, fell only a little short of the 90% threshold of SN/SP, with promising results to differentiate NMIBC from MIBC with a sensitivity of 75% and specificity of 73.7% (Taken et al. 2022). Future use of irisin in protein panels seems a viable option, however, this diagnostic method needs to be more researched to reach better results. Also, a recent meta-analysis connected Human papillomavirus (HPV) to a possibly higher susceptibility for BC and a worse prognosis (Sun et al. 2023). HPV is long connected to higher susceptibility and worse outcomes of cervical and penile cancers, it is only expected that the virus can travel through the urethra and affect the bladder (Okunade 2020; Iorga et al. 2020; Kidd et al. 2017). A study by Wang et al., on BC patients’ routine blood and urine parameters reported an association of higher urine pH with decreased risk of BC. Protein, glucose, and occult blood in urine showed an association with an increased risk of BC (Wang et al. 2021). Singh et al. reported elevated serum HMGB-1 levels in UBC patients with a positive correlation with disease severity and clinicopathological parameters (Singh et al. 2022). Aurora kinase and p53 family members expression studied in human BC cells showed, that in tumors with low p53 expression, the presence of either high AURKA or AURKB expression levels predicted an increased risk for relapse and mortality and high baseline AURKA expression predicted for inferior OS (Burgess et al. 2022). Bausch et al. investigated the association between urinary calprotectin and sterile leukocyturia in LG and HG BC, however, calprotectin did not qualify as a standalone marker, but rather a surrogate marker for tumor inflammation (Bausch et al. 2019).

Sangster et al. studied mutations in MIBC which is characterized by an abundance of mutations especially in chromatin modification and DNA damage response genes and reported two mutually exclusive mutation patterns in KDM6A and KMT2D as well as KDM6A and RB1, pointing to possible mutational interactions in BC (Sangster et al. 2022). A recent trial on the association of lncRNA and immunotherapy response including patients with BC showed that low and high scores of lncRNA differed significantly in overall survival, AUC on 12/20 months 0.79 and 0.77, respectively (Yu et al. 2020). Earlier this year, HLA-A*03 was associated with reduced overall survival after treatment with immune checkpoint inhibitors in the JAVELIN Solid Tumour trial (Naranbhai et al. 2022). The proposition for a novel BC biomarker is the 50 bp insertion/deletion polymorphism in superoxide dismutase (SOD1), where the SOD1 50-bp Ins/Del genotype, as well as Del, allele, was positively correlated with an elevated risk for BC in the Iranian population (Sarabandi et al. 2022).

In conclusion, the field of protein biomarkers is a vast one, studied for many decades either by histological staining or IHC methods. In recent years, gene expressions, epigenetic modifications, glycosylation, and analysis of extracellular vesicles (EVs) or non-coding RNA (ncRNA) have become the preferred and much-anticipated research standard. Various biomarkers have been identified using these methods, trying to make the diagnostic process more specific (Repiska et al. 2010). For BC, we chose the ones in the main text based on their significance and impact on BC diagnosis, recurrence, and progression. As seen in our review, the field of biomarkers is also a fast-growing one, fueled by rising cancer prevalence and the many issues of its diagnostics and therapy. Establishing tests and diagnostic methods to better and earlier determine cancer susceptibility, incidence, progression, or recurrence is an uneasy quest; however, a necessary one, for despite our best intentions, the incidence rises. Many patients ignore screening and advanced stages are harder or impossible to treat, while the number of specialists is also finite.
